# Association Between the Types of Alcoholic Beverages and Pancreatitis: A Cross-Sectional Study in a Tertiary Care Centre in Central India

**DOI:** 10.7759/cureus.79329

**Published:** 2025-02-19

**Authors:** Kartik Sharma, Pravin D Bhingare, Anup Wakodkar, Pankaj S Tongse, Pradeep S Shivsharan, Abhishek Rathod, Vaibhav Pandhare, Nikita S Monteiro, Harshil Rohit

**Affiliations:** 1 Department of General Surgery, Government Medical College Nagpur, Nagpur, IND

**Keywords:** acute interstitial pancreatitis, acute necrotising pancreatitis, acute pancreatitis, alcoholic beverages, daily drinking, desi daru/indian made indian liquor, distilled alcohol

## Abstract

Introduction

Alcohol, a psychoactive substance, affects the pancreas via multiple pathways and causes acute pancreatitis. Various types of alcoholic beverages are available in the market for consumption. This study aims to evaluate the association of the types of alcoholic beverages, the frequency of drinking, years of intake with presentation, and the type of alcohol-induced acute pancreatitis.

Methods

We conducted a cross-sectional study on 100 patients admitted to the Department of General Surgery, Government Medical College and Hospital (GMCH) Nagpur, a tertiary care centre in Central India, as cases of alcohol-induced acute pancreatitis over a period of six months from January 2024 to June 2024. Patients below 12 years of age, those diagnosed with other causes of acute pancreatitis, or those not willing to participate were excluded from the study.

Results

The present study indicates a higher prevalence of alcohol-induced acute pancreatitis among males, those in elementary occupations, and drinkers of Desi Daru/Indian-made Indian liquor (IMIL). Most patients started drinking between 16 and 30 years of age. The most common drinking pattern is daily, followed by an alternate-day drinking pattern. Most patients consume 5-10 drinks per occasion. Most patients developed symptoms after 5-10 years of alcohol consumption. Most patients presented to the hospital on the first episode of symptoms. Most patients had two to five episodes of symptoms. Most patients complain of epigastric pain, nausea/vomiting, and abdominal distension on first and repeat presentations. Desi Daru/IMIL drinkers more commonly develop acute necrotising pancreatitis on first and repeat presentations, whereas beer, wine, distilled, or mixed alcohol drinkers more commonly develop acute interstitial pancreatitis on first and repeat presentations.

Drinking patterns are found to be statistically significantly related to occupation groups and average standard drinks consumed per occasion. The types of alcoholic beverages are statistically significantly related to occupation groups, drinking patterns, and average intake years but not to the average standard drinks consumed per occasion or the number of symptomatic episodes or admissions. During the first presentation, the type of pancreatitis is statistically significantly related to the type of alcoholic beverages and drinking patterns but not to the average intake years or average drinks consumed per occasion. On repeat presentation, the type of pancreatitis is not statistically significantly related to the type of alcoholic beverages, drinking patterns, average intake years, or average standard drinks consumed per occasion.

Conclusion

This study concludes that the types of alcoholic beverages consumed are statistically significantly related to occupation groups, drinking patterns, and average intake years. Drinking patterns are also statistically significantly associated with occupation groups and the average standard drinks consumed per occasion.

During the first presentation, the type of pancreatitis is statistically significantly related to the type of alcoholic beverages and drinking pattern. Daily drinking of Desi Daru/IMIL has a higher chance of developing acute necrotising pancreatitis, whereas daily drinking of distilled alcohol has a higher chance of developing acute interstitial pancreatitis on initial presentation.

## Introduction

Alcohol (or ethanol by its chemical name) is a psychotropic substance produced by humans for a variety of uses, including consumption as beverages. Alcoholic drinks are a major source of energy [1]. Alcohol gets absorbed from the stomach and small intestine. The rate of absorption is quickest when alcohol is drunk on an empty stomach and the concentration of alcohol is 20-30% [1]. It is distributed throughout the water in the body so that most organs, such as the heart, brain, and muscles, are exposed to the same concentration of alcohol as found in the blood. The exception is the liver, where exposure is greater because blood is received directly from the stomach and small bowel via the portal vein. Alcohol diffuses rather slowly, except into organs with a rich blood supply, such as the brain and lungs. More than 90% of alcohol is removed by the liver, and 2-5% is excreted unaltered in urine, sweat, or breath [1]. Alcohol affects almost all organ systems (especially the central nervous system and hepatobiliary system) and causes many diseases, both directly and indirectly, including road traffic accidents and various cancers. One such disease is alcohol-induced pancreatitis, encompassing a spectrum from mild self-limiting acute pancreatitis to severe acute necrotising pancreatitis with multiple organ dysfunction syndrome.

There are two basic types of alcoholic beverages: distilled and undistilled. Undistilled beverages are prepared by fermentation of sugars (in grapes, wheat, barley, rice, and other grains) into ethanol by yeast. Examples of undistilled beverages are wine, beer, and hard cider. Distilled beverages are prepared by fermentation followed by distillation (the process by which alcohol concentration is increased in fermented substances). Examples of these are gin, brandy, whiskey, rum, tequila, and vodka.

In this study, we examine the association between the types of alcoholic beverages, drinking patterns, average drinks consumed per occasion, average intake years, and clinical presentation, as well as the type of pancreatitis.

## Materials and methods

We conducted a cross-sectional study of patients admitted to the Department of General Surgery, GMCH (Government Medical College and Hospital) Nagpur, as a case of alcohol-induced acute pancreatitis over a period of six months from January 2024 to June 2024.

Inclusion criteria

Patients who fulfilled two of the following criteria were diagnosed with acute pancreatitis: either abdominal pain consistent with acute pancreatitis (acute onset of a persistent, severe, epigastric pain often radiating to the back) or a threefold or higher elevation of serum lipase levels above the upper laboratory limit of normal, or characteristic findings of pancreatitis by imaging. Along with this, the patient should have a history of alcohol abuse, including at least one binge drinking episode [2] and should be willing to participate in the study.

Exclusion criteria

Patients below 12 years of age, patients not diagnosed with alcohol-induced acute pancreatitis, i.e., other causes of acute pancreatitis (gallstone-induced, ERCP (endoscopic retrograde cholangiopancreatography)-induced, drug-induced, trauma-induced), and patients not willing to participate in the study are excluded.

Parameters assessed

Demographic details (age, sex, profession) are collected. Profession is classified according to the International Standard Classification of Occupations (ISCO-08) major groups as 0 for armed forces occupations, 1 for managers, 2 for professionals, 3 for technicians and associate professionals, 4 for clerical support workers, 5 for service and sales workers, 6 for skilled agricultural, forestry, and fishery workers, 7 for craft and related trades workers, 8 for plant and machine operators and assemblers, and 9 for elementary occupations [3].

The type of alcohol consumed (Desi Daru/Indian-made Indian liquor (IMIL), beer, wine, distilled, mixed predominantly undistilled, mixed predominantly distilled) was inquired about. Patterns of alcohol intake (daily, alternate day, more than one per week, one per week, more than one per month, one per month, occasional drinking, single time) were also investigated, along with the age at which drinking was started and years of intake of alcoholic beverages.

The amount of alcohol consumed at a given time, in terms of standard drinks, was investigated. According to NIAAA (National Institute of Alcohol Abuse and Alcoholism), one standard drink or one alcoholic drink equivalent contains approximately 14 g of pure alcohol, which is found in 12 oz/354.9 mL of beer (containing 5% alcohol), 5 oz/147.9 mL of wine (containing 12% alcohol), and 1.5 oz/44.4 mL of distilled spirits (containing about 40% alcohol) [4]. As Desi Daru/IMIL's alcohol content varies a lot from 28% to 70%, no current standard definition is available. For uniform calculation, Desi Daru is considered to contain 40% alcohol.

The first episode of symptoms that occurred after the initiation of alcohol consumption was also inquired about, as well as the first presentation to any medical facility after initiating alcohol consumption requiring intravenous medications for symptomatic relief. Symptoms of first presentation to any medical facility (epigastric pain, nausea and vomiting, abdominal distension, fever) were recorded, as well as lipase levels at first presentation (not done, done but not known, <60 IU/L: normal, <200 IU/L: mildly raised, >200 IU/L: raised, >600 IU/L: high value).

The imaging findings at the first presentation (not done, done but not known, acute interstitial pancreatitis, acute on chronic pancreatitis, acute necrotising pancreatitis) and the number of episodes from the first presentation to the current presentation, including the first presentation (one, less than five, more than five, more than 10), were also inquired about. Additionally, symptoms of recurrent presentation to GMCH Nagpur (epigastric pain, nausea and vomiting, abdominal distension, and fever) were also inquired about.

Lipase levels at recurrent presentation to GMCH Nagpur (<60 IU/L: normal, <200 IU/L: mildly raised, >200 IU/L: raised, >600 IU/L: high value) were measured. Radiological imaging at a recurrent presentation at GMCH Nagpur (acute interstitial pancreatitis, acute on chronic pancreatitis, acute necrotising pancreatitis, chronic pancreatitis) was also conducted.

Sample size calculation

Assuming that outcome variable measures should be binary (alive/dead) and the possibility of success in each trial is p, the possibility of failure is 1-p, and the sampling distribution of the sample proportion is approximated to normal. Applying the formula, $n = Z^{2}_{1-\alpha/2}P(1-P)/d^{2}$, where n = sample size, $Z^{2}_{1-\alpha/2}$ = confidence interval, P = estimated precision, d = desired precision.

According to Sadr Azodi et al.'s study [5], the percentage of patients who developed acute pancreatitis due to alcohol (p) = 55.6%, absolute precision (d) = 10%, and desired confidence interval $1-\alpha/2$ = 95%. Thus, the minimum required sample size is 95.

Statistical analysis

Data were collected as per performa. Multifactorial statistical analysis was done using Microsoft Excel (Microsoft Corporation, Redmond, Washington). The continuous variables are presented as means and standard deviations. The categorical variables are presented as frequencies and percentages. The chi-square (Χ^2^) test was used for statistical analysis. A p-value of less than or equal to 0.05 is considered statistically significant. A p-value of less than or equal to 0.01 is considered statistically highly significant.

## Results

A total of 162 patients were admitted to the surgery department in GMCH Nagpur with complaints of acute pancreatitis, out of which 104 were alcohol-induced and 58 were due to other causes. Out of 104 remaining, three patients were unwilling to participate in the study, and one was below 12. Therefore, 100 patients were included in this study.

**Figure 1:**
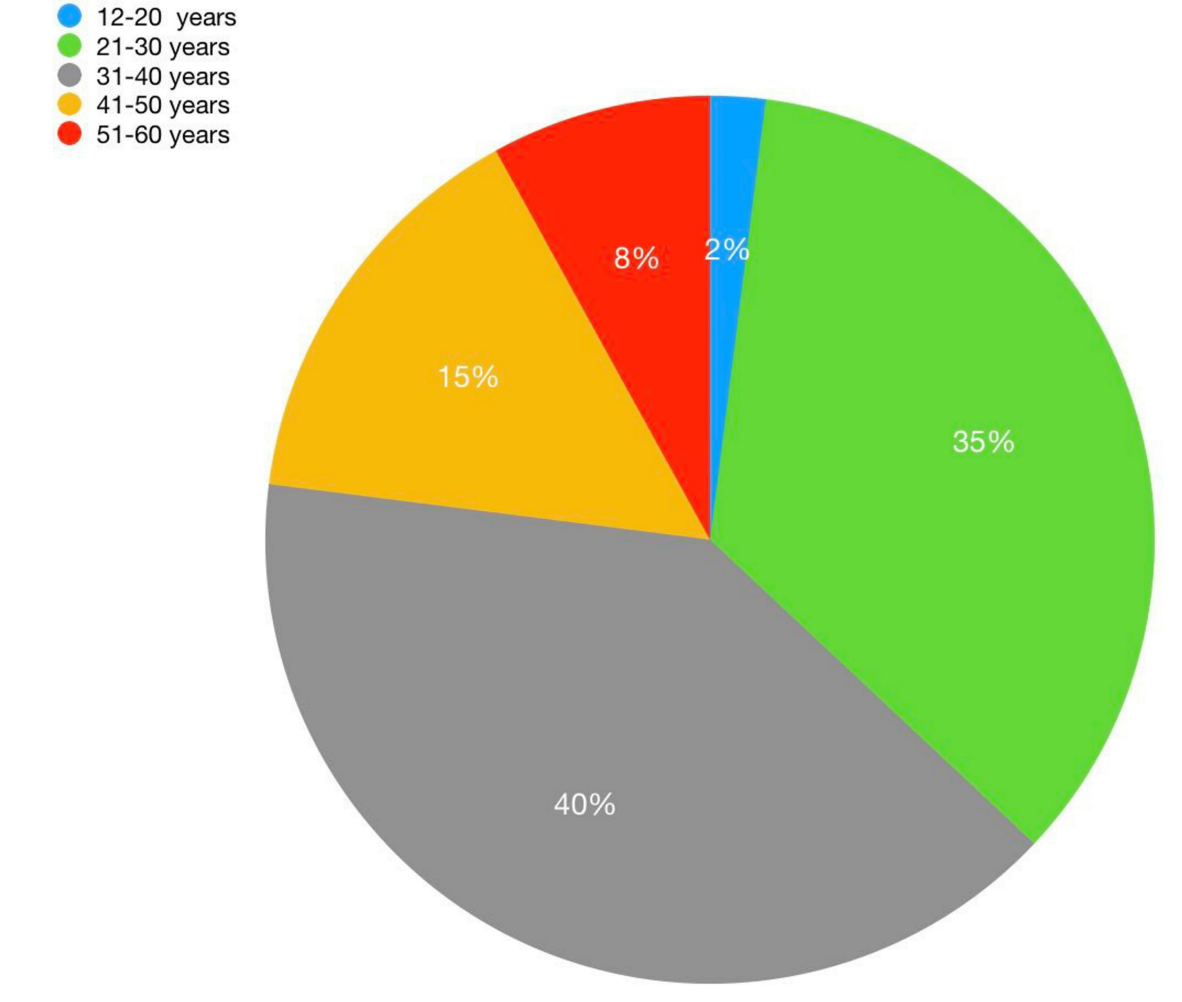
Age distribution of the patients

Out of 100 patients, 98 (98%) are males, and two (2%) are females. The age distribution of the participants is presented in Figure 1. The mean age of presentation was 34.33 years, with a standard deviation of 8.82 years. Most of the patients (40, 40%) belong to the 31-40 years age group, followed by 35 (35%) patients in the 21-30 years age group, followed by 15 (15%) patients in the 41-50 years age group.

**Figure 2:**
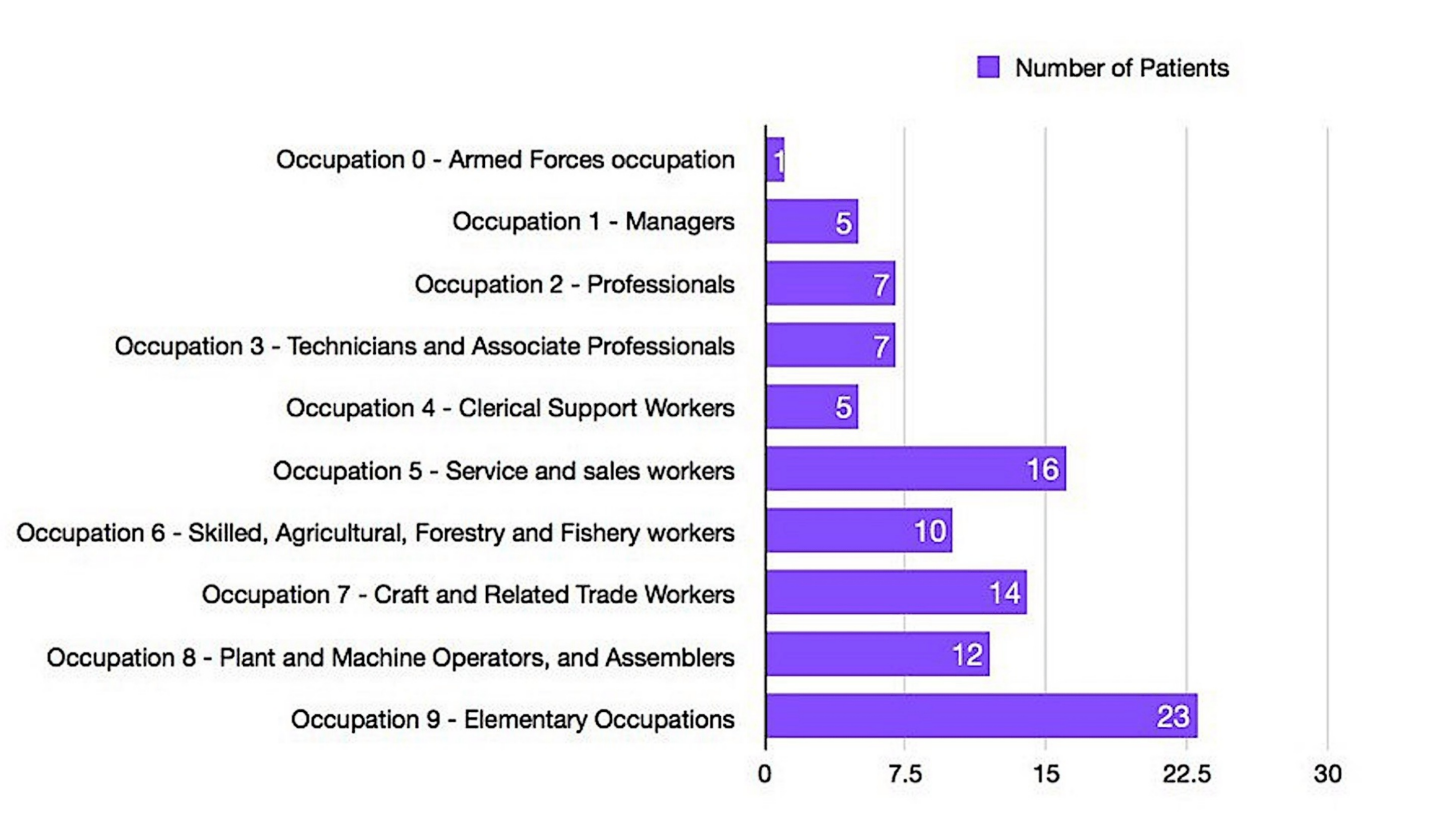
Occupation distribution of the patients

Figure 2 presents the occupation distribution of the participants. Most of the patients (23, 23%) belong to occupation group 9 (elementary occupations), followed by 16 (16%) belonging to occupation group 5 (service and sales workers). The lowest number of patients (one, 1%) belongs to occupation group 0 (armed forces occupation).

Types of alcoholic beverages consumed among various occupational groups are presented in Table 1. Most of the patients (41, 41%) included in this study drink Desi Daru/IMIL, followed by 24 (24%) patients drinking mixed (predominantly distilled) liquor. Desi daru/IMIL is preferred by 21 (21%) patients of the occupation group 9 (elementary occupations), followed by eight (8%) patients of the occupation group 7 (craft and related trade workers). Beer is consumed mostly by four (4%) patients of occupation group 5 (service and sales workers). Wine is preferred by two (2%) patients of occupation group 2 (professionals). Distilled alcoholic beverages are consumed mostly by four (4%) patients of occupation group 5 (service and sales workers). Occupation groups 6, 7, and 9 prefer Desi Daru/IMIL among other alcoholic beverages, accounting for five (5%), eight (8%), and 21 (21%) patients, respectively, while occupation group 8 prefers mixed (predominantly distilled) beverages, accounting for five (5%) patients. Applying the chi-square test, the X^2^ value is 107.41 with a degree of freedom of 45 (p<0.001), suggesting a statistically highly significant relation between the type of alcoholic beverages and occupation groups in acute pancreatitis patients.

**Table 1:** Type of alcoholic beverages consumed among various occupation major groups

Occupation Major Groups	Desi Daru/Indian-Made Indian Liquor (IMIL)	Beer	Wine	Distilled	Mixed (Predominantly Distilled)	Mixed (Predominantly Undistilled)	Total
0 - Armed Forces Occupations	0	0	0	1	0	0	1
1 - Managers	0	1	1	3	0	0	5
2 - Professionals	0	1	2	3	0	1	7
3 - Technicians and Associate Professionals	1	0	0	2	4	0	7
4 - Clerical Support Workers	1	1	0	1	0	2	5
5 - Service and Sales Workers	1	4	0	4	5	2	16
6 - Skilled, Agricultural, Forestry and Fishery Workers	5	0	0	1	3	1	10
7 - Craft and Related Trade Workers	8	0	0	0	5	1	14
8 - Plant and Machine Operators and Assemblers	4	1	0	2	5	0	12
9 - Elementary Occupations	21	0	0	0	2	0	23
Total	41	8	3	17	24	7	100

**Figure 3:**
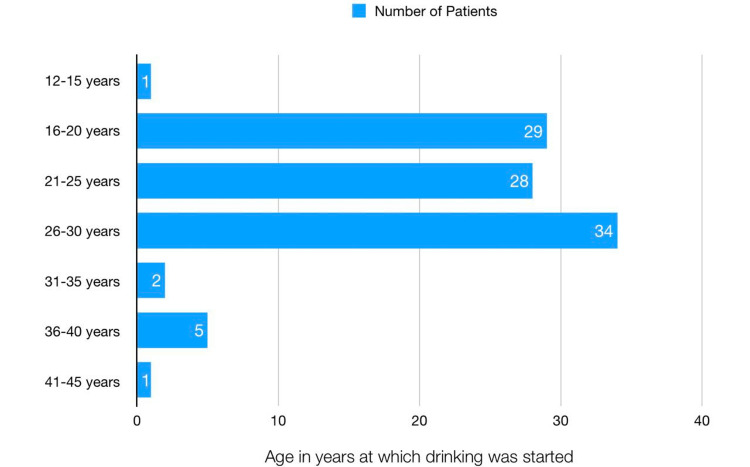
Age in years at which drinking alcoholic beverages was started

The age at which drinking was started is presented in Figure 3. The mean age of starting alcohol is 24.58 years with a standard deviation of 5.3 years. A total of 91 (91%) patients started drinking between the ages of 16 and 30 years, in which the most common age group is 26-30 years in 34 (34%) patients, followed by 16-20 years in 29 (29%) patients and followed by 21-25 years in 28 (28%) patients.

The pattern of alcohol consumed among various occupations in major groups is presented in Table 2. The most common pattern is daily in 58 (58%) patients, followed by alternate-day drinking in 24 (24%) patients. Almost in all occupation groups (except groups 0 and 5), the most common drinking pattern is Daily, whereas the most common drinking pattern is alternate-day drinking in Groups 0 and 5. Applying the chi-square test, the X^2^ value is 90.27 with a degree of freedom of 63 (p=0.013), suggesting a statistically significant relation between the pattern of alcoholic beverage consumption and occupation groups in acute pancreatitis patients.

**Table 2:** Drinking patterns among various occupation major groups

Occupation Major Groups	0	1	2	3	4	5	6	7	8	9	Total
Daily	0	2	2	3	2	6	7	9	9	18	58
Alternate-day drinking	1	1	0	2	1	9	2	4	1	3	24
>1/week	0	1	2	0	0	0	0	0	1	1	5
1/week	0	0	0	1	0	0	0	0	1	0	2
>1/month	0	0	0	0	1	0	0	1	0	0	2
1/month	0	0	1	0	0	0	1	0	0	1	3
Occasional	0	1	2	1	0	0	0	0	0	0	4
Single time	0	0	0	0	1	1	0	0	0	0	2
Total	1	5	7	7	5	16	10	14	12	23	100

The pattern of alcohol consumed in relation to the type of alcoholic beverages is presented in Table 3. The most common drinking pattern is daily in 58 (58%) patients, followed by alternate-day drinking in 24 (24%) patients. Patients with pancreatitis who consume Desi Daru/IMIL, beer, wine, distilled, or mixed (predominantly distilled) most commonly consume it daily, followed by alternate-day drinking, while those consuming mixed alcoholic beverages predominantly undistilled consume it most commonly on alternate days. Applying the chi-square test, the X^2^ value is 70.498 with a degree of freedom of 35 (p<0.001), suggesting a statistically highly significant relation between the type of alcoholic beverages and drinking patterns in acute pancreatitis patients.

**Table 3:** Drinking pattern/consumption frequency across various types of alcoholic beverages IMIL: Indian-made Indian liquor

Drinking Patterns of Alcoholic Beverages	Desi Daru/IMIL	Beer	Wine	Distilled	Mixed Predominantly Distilled	Mixed Predominantly Undistilled	Total
Daily	32	4	2	7	12	1	58
Alternate day	7	1	0	4	8	4	24
>1/week	1	0	0	2	1	1	5
1/week	0	0	0	1	1	0	2
>1/month	0	0	0	0	1	1	2
1/month	1	1	0	0	1	0	3
Occasional	0	0	1	3	0	0	4
Single time	0	2	0	0	0	0	2
Total	41	8	3	17	24	7	100

The average years of intake for the first episode of symptoms in relation to the type of alcohol consumed are presented in Table 4. The most common average alcohol intake years before the first episode of symptoms is 5-10 years in 50 (50%) patients, followed by less than five years in 31 (31%) patients. The most common average alcohol intake years before the first episode of symptoms in Desi Daru/IMIL, beer, mixed predominantly distilled, and mixed predominantly undistilled alcohol categories is also 5-10 years, whereas in the distilled alcohol category, it is less than five years. Two (2%) patients developed symptoms after a single episode of alcohol consumption, seen in the beer category only. Applying the chi-square test, the X^2^ value is 47.37 with a degree of freedom of 30 (p=0.023), suggesting a statistically significant relation between the type of alcoholic beverages and the average years of intake in acute pancreatitis patients.

**Table 4:** Average intake years for the first episode of symptoms in relation to the types of alcoholic beverages IMIL: Indian-made Indian liquor

Average Years of Intake	Desi Daru/IMIL	Beer	Wine	Distilled	Mixed Predominantly Distilled	Mixed Predominantly Undistilled	Total
Single episode	0	2	0	0	0	0	2
< 5 years	12	1	1	8	6	3	31
5–10 years	21	5	1	7	12	4	50
11–15 years	4	0	0	1	1	0	6
16–20 years	1	0	1	0	4	0	6
21–25 years	3	0	0	0	1	0	4
> 25 years	0	0	0	1	0	0	1
Total	41	8	3	17	24	7	100

The average number of standard drinks consumed per occasion in relation to various alcohol consumed is shown in Table 5. The most common number of average standard drinks consumed per occasion is 5-10 drinks in 39 (39%) patients, followed by less than five drinks in 37 (37%) patients. In Desi Daru/IMIL, beer, and mixed predominantly distilled alcohol categories, the most common number of average standard drinks consumed is 5-10 drinks, whereas in distilled and mixed predominantly undistilled alcohol categories, the most common number of standard drinks consumed is less than five drinks. Applying the chi-square test, the X^2^ value is 11.35 with a degree of freedom of 15 (p=0.73), suggesting no statistically significant relation between the type of alcoholic beverages and average standard drinks consumed per occasion in acute pancreatitis patients.

**Table 5:** Average standard drinks consumed per occasion in relation to the type of alcoholic beverages IMIL: Indian-made Indian liquor

Average Standard Drinks Consumed per Occasion	Desi Daru/IMIL	Beer	Wine	Distilled	Mixed Predominantly Distilled	Mixed Predominantly Undistilled	Total
<5 drinks	13	3	1	9	8	3	37
5–10 drinks	14	5	1	6	11	2	39
11–15 drinks	11	0	1	2	5	2	21
>15 drinks	3	0	0	0	0	0	3
Total	41	8	3	17	24	7	100

The average number of standard drinks consumed per occasion in relation to drinking patterns is presented in Table 6. The most common number of standard drinks consumed per occasion in daily, alternate-day, and single-time consumption categories is 5-10 drinks in 25 (25%), 11 (11%), and two (2%) patients, respectively, whereas in more than one per week, one per week, more than one per month, one per month, and occasional consumption categories, it is less than five drinks in four (4%), two (2%), two (2%), and two (2%) patients, respectively. Applying the chi-square test, the X^2^ value is 37.83 with a degree of freedom of 21 (p=0.048), suggesting a statistically significant relation between drinking patterns and average standard drinks consumed per occasion in acute pancreatitis patients.

**Table 6:** Average standard drinks consumed per occasion in relation to drinking pattern/consumption frequency

Average Standard Drinks Consumed per Occasion	<5 drinks	5–10 drinks	11–15 drinks	>15 drinks	Total
Daily	16	25	15	2	58
Alternate day	9	11	4	0	24
>1/week	4	1	0	0	5
1/week	2	0	0	0	2
>1/month	2	0	0	0	2
1/month	2	0	0	1	3
Occasional	2	0	2	0	4
Single time	0	2	0	0	2
Total	37	39	21	3	100

**Figure 4:**
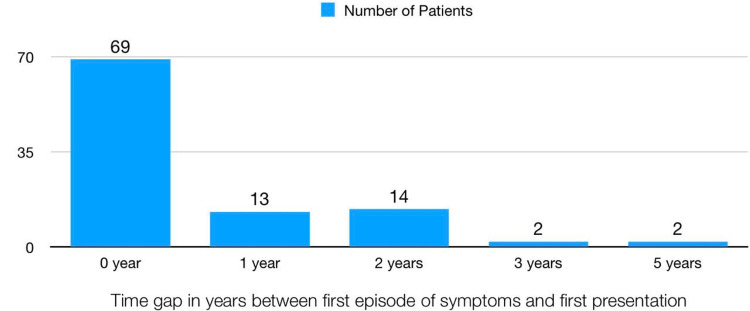
Time difference in years between the first episode of symptoms and the first presentation to a healthcare facility

The difference in years between the first episode of symptoms and the first presentation is shown in Figure 4. The mean years of difference between the first episode of symptoms and the first presentation is 0.57, with a standard deviation of 0.99 years. Most patients presented to the hospital at their first episode of symptoms (i.e., at zero year difference) in 70 (70%) patients, followed by two years of difference in 14 (14%) patients.

The symptoms of the first presentation to any healthcare facility in relation to the type of alcohol consumed are presented in Table 7. Most patients presented with epigastric pain in 95 (95%) patients, followed by nausea/vomiting in 51 (51%) patients, followed by abdominal distension in 28 (28%) patients. The pattern is also the same in individual alcohol categories. Applying the chi-square test, the X^2^ value is 33.97 with a degree of freedom of 30 (p=0.28), suggesting no statistically significant relation between the type of alcoholic beverage and symptoms at first presentation in acute pancreatitis patients.

**Table 7:** Symptoms at first presentation in relation to the type of alcoholic beverages IMIL: Indian-made Indian liquor

Symptoms at First Presentation	Desi Daru/IMIL	Beer	Wine	Distilled	Mixed Predominantly Distilled	Mixed Predominantly Undistilled	Total
Epigastric pain	40	7	3	16	22	7	95
Nausea/vomiting	33	3	1	4	9	1	51
Hematemesis	0	1	0	0	0	0	1
Abdominal distension	21	1	0	1	4	1	28
Fever	2	0	0	0	0	0	2
Jaundice	2	0	0	0	0	0	2
Altered sensorium	2	0	0	0	0	0	2
Total	41	8	3	17	24	7	100

The lipase value at first presentation in relation to alcohol consumed is presented in Table 8. Most patients had high lipase values at first presentation in 40 (40%), followed by raised lipase values in 33 (33%). Most patients drinking Desi Daru/IMIL, wine, or mixed predominately undistilled had high lipase values at first presentation in 22 (22%), two (2%), and two (2%) patients, respectively, whereas most patients drinking beer and distilled alcohols had raised lipase values at first presentation in three (3%) and 10 (10%) patients, respectively. In six (6%) patients, the lipase level was not measured at first presentation, while lipase levels were measured but not known in 18 (18%) patients. Applying the chi-square test, the X^2^ value is 26.08 with a degree of freedom of 20 (p=0.16), suggesting no statistically significant relation between the type of alcoholic beverage and lipase value at primary presentation.

**Table 8:** Lipase values at first presentation in relation to the type of alcoholic beverages IMIL: Indian-made Indian liquor

Lipase at First Presentation	Desi Daru/IMIL	Beer	Wine	Distilled	Mixed Predominantly Distilled	Mixed Predominantly Undistilled	Total
Not done	2	0	0	0	2	2	6
Done but not known	6	2	0	3	6	1	18
Mildly raised	1	1	0	0	0	1	3
Raised	10	3	1	10	8	1	33
High value	22	2	2	4	8	2	40
Total	41	8	3	17	24	7	100

The imaging findings at the first presentation in relation to alcohol consumed are shown in Table 9. The most common imaging finding at first presentation was acute interstitial pancreatitis in 44 (44%) patients, followed by acute necrotising pancreatitis in 28 (28%) patients. Most patients drinking Desi Daru/IMIL presented with acute necrotising pancreatitis at their first presentation in 23 (23%) patients, whereas most patients drinking beer, wine, distilled, mixed predominantly distilled, and mixed predominantly undistilled alcohol presented with acute interstitial pancreatitis in five (5%), three (3%), 11 (11%), and three (3%) patients, respectively. Conversely, patients presenting with acute necrotising pancreatitis at first presentation consumed Desi Daru/IMIL most commonly in 23 (82.1%) patients, while patients presenting with acute interstitial pancreatitis consumed distilled alcohol most commonly in 14 (31%) patients, followed by mixed predominantly distilled in 11 (25%) patients. Applying the chi-square test, the X^2^ value is 52.674 with a degree of freedom of 25 (p<0.001), suggesting a statistically highly significant relation between the type of alcoholic beverages and the type of pancreatitis at first presentation.

**Table 9:** Type of pancreatitis at first presentation in relation to the type of alcoholic beverages IMIL: Indian-made Indian liquor

Imaging Finding at First Presentation	Desi Daru/IMIL	Beer	Wine	Distilled	Mixed Predominantly Distilled	Mixed Predominantly Undistilled	Total
Not done	4	0	0	0	6	2	12
Done but not known	5	2	0	3	1	1	12
Acute interstitial pancreatitis	8	5	3	14	11	3	44
Acute necrotising pancreatitis	23	1	0	0	3	1	28
Acute on chronic pancreatitis	1	0	0	0	2	0	3
Chronic pancreatitis	0	0	0	0	1	0	1
Total	41	8	3	17	24	7	100

The pattern of alcoholic beverages consumed in relation to the type of pancreatitis at first presentation is presented in Table 10. The most common drinking pattern is daily in 58 (58%) patients, followed by alternate days in 24 (24%) patients. All types of acute pancreatitis at first presentation are more common in daily drinking patterns, followed by alternate-day drinking patterns. Applying the chi-square test, the X^2^ value is 49.76 with a degree of freedom of 35 (p=0.05), suggesting a statistically significant relation between the drinking patterns and the type of pancreatitis on the first presentation.

**Table 10:** Drinking patterns/consumption frequency in relation to the type of pancreatitis at first presentation

Drinking Patterns of Alcoholic Beverages	Not Done	Done but Not Known	Acute Interstitial Pancreatitis	Acute Necrotising Pancreatitis	Acute on Chronic Pancreatitis	Chronic Pancreatitis	Total
Daily	6	10	17	23	2	0	58
Alternate day	4	2	12	5	1	0	24
>1/week	0	0	4	0	0	1	5
1/week	1	0	1	0	0	0	2
>1/month	1	0	1	0	0	0	2
1/month	0	0	3	0	0	0	3
Occasional	0	0	4	0	0	0	4
Single time	0	0	2	0	0	0	2
Total	12	12	44	28	3	1	100

The average intake years of alcoholic beverages in relation to the type of pancreatitis at first presentation is presented in Table 11. The most common average alcohol intake years before the first episode of symptoms is 5-10 years in 50 (50%) patients, followed by less than five years in 31 (31%) patients. All types of pancreatitis are more common among 5-10 years of alcoholic drinkers, except acute on chronic pancreatitis. Applying the chi-square test, the X^2^ value is 30.472 with a degree of freedom of 30 (p=0.442), suggesting no statistically significant relation between average intake years of alcoholic beverage and the type of pancreatitis at first presentation.

**Table 11:** Average intake years in relation to the type of pancreatitis at first presentation

Average Years of Intake	Not Done	Done but Not Known	Acute Interstitial Pancreatitis	Acute Necrotising Pancreatitis	Acute on Chronic Pancreatitis	Chronic Pancreatitis	Total
Single episode	0	0	2	0	0	0	2
<5 years	5	2	14	9	1	0	31
5–10 years	6	10	21	12	0	1	50
11–15 years	0	0	3	3	0	0	6
16–20 years	1	0	3	1	1	0	6
21–25 years	0	0	0	3	1	0	4
>25 years	0	0	1	0	0	0	1
Total	12	12	44	28	3	1	100

The average standard drinks consumed per occasion in relation to the type of pancreatitis at first presentation are shown in Table 12. The most common number of average standard drinks consumed per occasion is 5-10 drinks in 39 (39%) patients, followed by less than five drinks in 37 (37%) patients. All types of acute pancreatitis are common in the less than five average standard drinks per occasion group. Applying the chi-square test, the X^2^ value is 9.944 with a degree of freedom of 15 (p=0.823), suggesting no statistically significant relation between the average standard drinks consumed per occasion and the type of pancreatitis.

**Table 12:** Average standard drinks consumed in relation to the type of pancreatitis at first presentation

Average Standard Drinks Consumed per Occasion	Not Done	Done but Not Known	Acute Interstitial Pancreatitis	Acute Necrotising Pancreatitis	Acute on Chronic Pancreatitis	Chronic Pancreatitis	Total
<5 drinks	4	6	20	6	1	0	37
5–10 drinks	5	5	15	12	1	1	39
11–15 drinks	3	1	8	8	1	0	21
>15 drinks	0	0	1	2	0	0	3
Total	12	12	44	28	3	1	100

The number of episodes, including the first and current episodes of symptoms, in relation to the type of alcohol consumed is presented in Table 13. Most patients had two to five episodes of symptoms in 50 (50%) patients, including the first and current episodes of symptoms. A total of 34 (34%) patients presented to the hospital at the first episode of symptoms. Applying the chi-square test, the X^2^ value is 12.773 with a degree of freedom of 15 (p=0.62), suggesting no statistically significant relation between the number of episodes of symptoms and the type of alcoholic beverages.

**Table 13:** The number of episodes of symptoms including the first and current presentations in relation to the type of alcoholic beverages IMIL: Indian-made Indian liquor

No. of Episodes Including the First and Current Presentations	Desi Daru/IMIL	Beer	Wine	Distilled	Mixed Predominantly Distilled	Mixed Predominantly Undistilled	Total
1st episode	14	3	2	6	8	1	34
2–5 episodes	16	5	1	10	12	6	50
6–10 episodes	10	0	0	1	4	0	15
>10 episodes	1	0	0	0	0	0	1
Total	41	8	3	17	24	7	100

The number of admissions, including the first and current admissions, in relation to the type of alcohol consumed is shown in Table 14. Most patients had two to five admissions, including the first and current presentations, in 57 (57%) patients. In almost all alcohol categories except wine, the two to five admissions group is the most common. A total of 39 (39%) patients were admitted for the first time. More than five admissions are more common in the Desi Daru/IMIL group in three (3%) patients than in the distilled group in one (1%) patient. Applying the chi-square test, the X^2^ value is 6.94 with a degree of freedom of 10 (p=0.73), suggesting no statistically significant relation between the number of admissions and the type of alcoholic beverages in acute pancreatitis patients.

**Table 14:** The number of admissions including the current presentation in relation to the type of alcoholic beverages IMIL: Indian-made Indian liquor

No. of Admissions Including the First and Current Presentations	Desi Daru/IMIL	Beer	Wine	Distilled	Mixed Predominantly Distilled	Mixed Predominantly Undistilled	Total
1st admission	18	3	2	6	9	1	39
2–5 admissions	20	5	1	10	15	6	57
6–10 admissions	3	0	0	1	0	0	4
Total	41	8	3	17	24	7	100

The symptoms on repeat presentation in relation to the type of alcohol consumed are presented in Table 15. A total of 34 (34%) patients presented to the hospital at the first episode of symptoms. Out of 66 repeat presentations, 66 (100%) patients complained of epigastric pain, making it the most common symptom on repeat presentation. The second most common symptom on repeat presentation is vomiting in 54 (81%) patients, followed by abdominal distension in 20 (30%) patients. Jaundice is also more common on repeat presentation in five (7.5%) patients than on the first presentation in two (2%) patients. Applying the chi-square test, the X^2^ value is 16.0 with a degree of freedom of 20 (p=0.71), suggesting no statistically significant relation between the type of alcoholic beverage and symptoms of repeat presentation in acute pancreatitis patients.

**Table 15:** Symptoms at repeat presentation in relation to the type of alcoholic beverages IMIL: Indian-made Indian liquor

Symptoms for Repeated Presentation	Desi Daru/IMIL	Beer	Wine	Distilled	Mixed Predominantly Distilled	Mixed Predominantly Undistilled	Total
Epigastric pain	27	5	1	11	16	6	66
Nausea/vomiting	23	4	0	10	12	5	54
Abdominal distension	14	1	0	1	4	0	20
Fever	0	0	0	1	0	0	1
Jaundice	3	1	0	0	1	0	5
Total	27	5	1	11	16	6	66

The lipase levels on repeat presentation in relation to alcohol consumed are presented in Table 16. Most patients on repeat presentation have high lipase values in 33 (50%) patients, followed by raised lipase values in 26 (39%) patients. High lipase values are more common than raised lipase values in all alcohol categories except beer and mixed predominantly undistilled categories. One (1.5%) patient presented with a normal lipase value on repeat presentation. Applying the chi-square test, the X^2^ value is 11.38 with a degree of freedom of 15 (p=0.72), suggesting no statistically significant relation between the type of alcoholic beverage and lipase levels.

**Table 16:** Lipase values on repeat presentation in relation to the type of alcoholic beverages IMIL: Indian-made Indian liquor

Lipase on Repeat Presentation	Desi Daru/IMIL	Beer	Wine	Distilled	Mixed Predominantly Distilled	Mixed Predominantly Undistilled	Total
Normal	1	0	0	0	0	0	1
Mildly raised	2	1	0	1	0	2	6
Raised	11	3	0	3	7	2	26
High value	13	1	1	7	9	2	33
Total	27	5	1	11	16	6	66

The imaging findings on repeat presentation in relation to alcohol consumed are presented in Table 17. Most patients have acute interstitial pancreatitis on repeat presentation in 28 (42%) patients, followed by acute necrotising pancreatitis in 19 (28%) patients, followed by acute on chronic pancreatitis in 18 (27%) patients. In the Desi Daru/IMIL category, acute necrotising pancreatitis in 15 (55%) patients is the most common imaging finding on repeat presentation, followed by acute on chronic pancreatitis in six (22%) and acute interstitial pancreatitis in five (18%) patients. In all other alcohol categories, acute interstitial pancreatitis is most common, followed by acute on chronic pancreatitis, followed by acute necrotising pancreatitis. Applying the chi-square test, the X^2^ value is 21.41 with a degree of freedom of 15 (p=0.124), suggesting no statistically significant relation between the type of alcoholic beverage and the type of pancreatitis on repeat presentation.

**Table 17:** Imaging findings/type of pancreatitis on repeat presentation in relation to the type of alcoholic beverages IMIL: Indian-made Indian liquor

Imaging Finding on Repeat Presentation	Desi Daru/IMIL	Beer	Wine	Distilled	Mixed Predominantly Distilled	Mixed Predominantly Undistilled	Total
Acute interstitial pancreatitis	5	2	1	7	9	4	28
Acute necrotising pancreatitis	15	1	0	1	2	0	19
Acute on chronic pancreatitis	6	2	0	3	5	2	18
Chronic pancreatitis	1	0	0	0	0	0	1
Total	27	5	1	11	16	6	66

The patterns of alcohol consumed in relation to the type of pancreatitis on repeat presentation are presented in Table 18. The most common drinking pattern in repeat presenters is daily in 41 (62%) patients, followed by alternate days in 18 (27.3%). All types of acute pancreatitis on repeat presentation are common in daily drinkers, followed by alternate-day drinkers. Applying the chi-square test, the X^2^ value is 11.8 with a degree of freedom of 15 (p=0.694), suggesting that there is no statistically significant relation between the drinking patterns of alcoholic beverages and the type of pancreatitis on repeat presentation.

**Table 18:** Drinking pattern/consumption frequency in relation to the type of pancreatitis on repeat presentation

Drinking Patterns of Alcoholic Beverages	Acute Interstitial Pancreatitis	Acute Necrotising Pancreatitis	Acute on Chronic Pancreatitis	Chronic Pancreatitis	Total
Daily	13	15	13	0	41
Alternate day	9	4	4	1	18
>1/week	3	0	1	0	4
1/week	1	0	0	0	1
>1/month	1	0	0	0	1
Occasional	1	0	0	0	1
Total	28	19	18	1	66

The average intake years of alcoholic beverages in relation to the type of pancreatitis on repeat presentation are presented in Table 19. Most patients on repeat presentation drank alcohol for an average of 5-10 years in 31 (47%) patients, followed by 11-15 years in 14 (21.2%) patients. All types of pancreatitis are most common in the average 5-10 years intake group. Applying the chi-square test, the X^2^ value is 14.43 with a degree of freedom of 15 (p=0.493), suggesting no statistically significant relation between the average intake years of alcoholic beverages and the type of pancreatitis on repeat presentation.

**Table 19:** Average intake years in relation to the type of pancreatitis on repeat presentation

Average Years of Intake	Acute Interstitial Pancreatitis	Acute Necrotising Pancreatitis	Acute on Chronic Pancreatitis	Chronic Pancreatitis	Total
<5 years	3	1	4	0	8
5–10 years	12	9	9	1	31
11–15 years	9	3	2	0	14
16–20 years	4	2	0	0	6
21–25 years	0	1	1	0	2
>25 years	0	3	2	0	5
Total	28	19	18	1	66

The average standard drinks consumed per occasion in relation to the type of pancreatitis on repeat presentation are presented in Table 20. Most patients on repeat presentation drank an average of 5-10 standard drinks per occasion in 31 (47%) patients, followed by less than five standard drinks per occasion in 21 (31.8%). All types of acute pancreatitis on repeat presentation are most common in the average 5-10 drinks per occasion group. Applying the chi-square test, the X^2^ value is 6.88 with a degree of freedom of nine (p=0.649), suggesting no statistically significant relation between the average standard drinks per occasion of alcoholic beverages and the type of pancreatitis on repeat presentation.

**Table 20:** Average standard drinks consumed in relation to the type of pancreatitis on repeat presentation

Average Standard Drinks Consumed per Occasion	Acute Interstitial Pancreatitis	Acute Necrotising Pancreatitis	Acute on Chronic Pancreatitis	Chronic Pancreatitis	Total
<5 drinks	11	4	5	1	21
5–10 drinks	12	10	9	0	31
11–15 drinks	5	5	3	0	13
>15 drinks	0	0	1	0	1
Total	28	19	18	1	66

The summary of the relationship between the various parameters studied is depicted in Table 21. Drinking patterns are found to be statistically significantly related to occupation groups and average standard drinks consumed per occasion. Types of alcoholic beverages are statistically significantly related to occupation groups, drinking patterns, and average intake years but not to the average standard drinks consumed per occasion or the number of symptomatic episodes or admissions. In the first presentation, the type of pancreatitis is statistically significantly related to the type of alcoholic beverages and drinking patterns but not to the average intake years or average drinks consumed per occasion. On repeat presentation, the type of pancreatitis is not statistically significantly related to the type of alcoholic beverages, drinking patterns, average intake years, or average standard drinks consumed per occasion.

**Table 21:** Summary of the association of parameters assessed and their level of statistical significance

Presentation	Association Between Parameters		P-values	Level of Statistical Significance
	Parameter 1	Parameter 2		
Both	Occupation groups	Drinking pattern	0.013	Significant
Both	Drinking pattern	Average standard drinks consumed per occasion	0.048	Significant
Both	Type of alcoholic beverage	Occupation group	< 0.001	Highly significant
		Drinking pattern	< 0.001	Highly significant
		Average intake years	0.023	Significant
		Average standard drinks consumed per occasion	0.73	Not significant
		No. of episodes of symptoms	0.62	Not significant
		No. of admissions	0.73	Not significant
First	Type of alcoholic beverage	Symptoms at presentation	0.28	Not significant
		Lipase value	0.16	Not significant
First	Type of pancreatitis	Type of alcoholic beverage	< 0.001	Highly significant
		Drinking pattern	0.05	Significant
		Average intake years	0.44	Not significant
		Average standard drinks consumed per occasion	0.82	Not significant
Repeat	Type of alcoholic beverage	Symptoms at presentation	0.71	Not significant
		Lipase value	0.72	Not significant
Repeat	Type of pancreatitis	Type of alcoholic beverage	0.12	Not significant
		Drinking pattern	0.69	Not significant
		Average intake years	0.49	Not significant
		Average standard drinks consumed per occasion	0.65	Not significant

## Discussion

Acute pancreatitis is caused mainly by alcohol abuse, gallstones, anatomical obstruction, ERCP, drugs, hypercalcemia, hypertriglyceridemia, trauma, infections, and hypotension. Almost 60-80% of acute pancreatitis cases in developed countries are caused by gallstone disease or alcohol abuse [6]. The pathophysiology of alcohol-induced acute pancreatitis is complicated and predominantly includes increased oxidative stress [7,8], disruption of cytosolic calcium homeostasis [9], and changes in gene expression [10] in the pancreas.

Acute pancreatitis is mild and resolves itself without serious complications in 80% of patients. Morbidity and mortality occur in up to 20% of patients despite the aggressive intervention [11]. This is usually due to SIRS (systemic inflammatory response syndrome) and organ failure in the first two-week period. In contrast, after two weeks, it is usually due to sepsis and its complications [12]. In a systematic review of studies of acute pancreatitis, the overall mortality was approximately 5%. Mortality rates in patients with interstitial and necrotising pancreatitis were 3% and 17%, respectively [13].

There are specific patterns of alcohol consumption, outlined as follows: social drinking or moderate drinking refers to men not having more than two drinks per day and women not having more than one drink per day. It refers to the use of alcohol in a single day and not an average over several days [14]. Binge drinking refers to consuming five or more drinks by men and four or more drinks by women on a single occasion in approximately two hours [2]. A harmful drinking pattern refers to an alcohol consumption pattern that results in physical or psychological harm to the individual or society. This disorder is also recognised by the World Health Organisation (WHO) [15]. A hazardous drinking pattern is defined as a quantity or pattern of alcohol consumption that places individuals at risk for adverse health events [2]. Alcohol dependence is defined as a maladaptive pattern of drinking, leading to clinically significant impairment or distress, as manifested by three or more of the following occurring at any time in the same 12-month period: need for markedly increased amounts of alcohol to achieve intoxication or desired effect; or markedly diminished effect with continued use of the same amount of alcohol, the characteristic withdrawal syndrome for alcohol; or drinking (or using a closely related substance) to relieve or avoid withdrawal symptoms, drinking in larger amounts or over a longer period than intended, persistent desire or one or more unsuccessful efforts to cut down or control drinking, important social, occupational, or recreational activities given up or reduced because of drinking, a great deal of time spent in activities necessary to obtain, to use, or to recover from the effects of drinking, continued drinking despite knowledge of having a persistent or recurrent physical or psychological problem that is likely to be caused or exacerbated by drinking [16]. Alcohol use disorder (AUD) is a pattern of alcohol use that involves problems controlling your drinking, being preoccupied with alcohol, continuing to use alcohol even when it causes problems, having to drink more to get the same effect, or having withdrawal symptoms when rapidly decreased or stop drinking [17].

According to WHO, the total per capita consumption of alcohol by individuals above 15 years of age is 6.2 L of pure alcohol per year, which equals 13.5 g of pure alcohol per day. Nearly 5.1% of the global burden of disease is attributable to alcohol consumption, and it causes almost 3.3 million deaths every year [17]. The prevalence of AUDs is highest in Europe (7.5%) and lowest among eastern Mediterranean regions, which include Afghanistan, Bahrain, and Egypt [17]. The 12-month prevalence of AUDs in India in 2010 was 2.6% and that of alcohol dependence was 2.1% [18].

According to the National Family Health Survey (NFHS-5) conducted by the Ministry of Health and Family Welfare, Government of India, in 2019-2021, 18.7% of men and 1.3% of women above 15 years of age consume alcohol. Among men, alcohol is most commonly consumed by those 35-49 years of age, with no schooling, Christian religion, in the scheduled tribe, and in the lowest wealth quintile. Among women, alcohol is most commonly consumed by those 50-64 years of age, by those with no schooling, by those of Buddhist/Neo-Buddhist religion, by those of scheduled tribes, and by those in the lowest wealth quintile. According to NFHS-5, 13.9% of men and 0.4% of women above 15 years of age consume alcohol in Maharashtra, Central India [19].

In the current study, the percentage of males is found to be 98%, which is much higher compared to 65.4% in the Cohort of Swedish Men (COSM) study [5], a case-control study on the Japanese population [20], and 54.8% in North Indian males [21]. In contrast, the percentage of females is 2%, which is significantly lower compared to 34.4% in the Swedish Mammography Cohort (SMC) [5] and 45.1% in North Indian females [21]. This is because of the socio-cultural conditions of India that females are less involved in alcohol drinking.

In the current study, 30% of patients started drinking at less than 21 years, consistent with the rapidly changing trend of starting drinking at a younger age in India. The percentage of the drinking population younger than 21 years has increased from 2% to more than 14% in past years. The reasons contributing to this are the changing norms, urbanisation, increased availability, high-intensity mass marketing, and relaxation of overseas trade rules, along with a poor level of awareness related to the harmful effects of alcohol [22].

In the current study of alcohol-induced pancreatitis patients, most patients are employed in the occupation group 9 (elementary occupations, 23%), belong to the age group 31-40 years (40%), started drinking at age 26-30 years (34%), drink Desi Daru/IMIL (41%), drink daily (58%), drink about 5-10 drinks per occasion (39%), drink for 5-10 years (58%), have a history of two to five episodes of symptoms (50%), and have admissions (57%).

The mean age of patients is 34.33 years, with the majority in the 31-40 years age group (40%), which is comparable to the 39.21 years with the majority of the patients in the 31-40 years age group (28%) in the Karim et al. North Indian hospital study [21] was lower than the 57.6 years in the Kume et al. study on the Japanese population [20]. The mean years of alcohol intake for acute pancreatitis is 7.87 years, which is more compared to 5.1 years in the Swedish cohort [5].

In the current study, drinking patterns are found to be statistically significantly related to occupation groups and average standard drinks consumed per occasion. Types of alcoholic beverages are statistically significantly related to occupation groups, drinking patterns, and average intake years but not to the average standard drinks consumed per occasion or the number of symptomatic episodes or admissions.

The most common complaints of patients at first presentation in the current study were epigastric pain (95%), nausea/vomiting (51%) and abdominal distension (28%), which is comparable to pain in the abdomen (100%) and abdominal distension (29%) in the Karim et al. study of the North Indian population [21].

Desi Daru/IMIL drinking patients have the highest percentage of acute necrotising pancreatitis on first presentation (56%) and repeat presentation (55%). Patients drinking beer, wine, distilled, and mixed alcohol have a higher percentage of acute interstitial pancreatitis on the first and repeat presentations. Conversely, acute necrotising patients presenting for the first time are mostly Desi Daru/IMIL drinkers (82%), whereas acute interstitial pancreatitis patients presenting for the first time are mostly distilled (31%) or mixed predominantly distilled (25%) alcohol drinkers. The patients with acute necrotising pancreatitis as the index presentation was lower compared to 73.5% in Patra et al.'s study and 70.3% in Angelini et al.'s study and higher compared to 22.3% in Beger et al., 22% in Pelli et al., 18% in Ahmed Ali et al. [21].

In the current study, for the initial presentation, the type of pancreatitis is statistically significantly related to the type of alcoholic beverages and drinking patterns but not to the average intake years or average drinks consumed per occasion. In the Swedish cohort, there was a 52% significantly increased risk of acute pancreatitis with every increase of five standard drinks in the amount of spirits (distilled alcohol) consumed on a single occasion (dose-response association), but not with the amount of wine or beer. Furthermore, there was no association between the risk of acute pancreatitis and the consumption frequency of different alcoholic beverages or the average amount of alcohol consumed per month [5]. In the Danish population, the development of acute and chronic pancreatitis is increased with alcohol intake (J-shaped association), with a significant increase among abstainers, those drinking >14 drinks per week, those drinking beer and spirits, frequent binge drinkers and daily drinkers but not with wine consumers [23]. In a case-control study on the Japanese population by Kume et al., there was an increased but non-linear association between the amount of alcohol intake per day and the incidence of acute pancreatitis and the most increased risk of total pancreatitis when drinking whiskey (distilled) and wine [20]. A cross-sectional study in the Indian population showed that binge drinking was more frequent among patients with alcoholic pancreatitis than those with alcoholic liver disease [22]. Meta-analyses have also shown that alcohol intake of more than two to four drinks per day is associated with an increased risk of pancreatitis [24-27].

In the current study, for repeat presentation, statistically, the type of pancreatitis is not significantly related to the type of alcoholic beverages, drinking patterns, average intake years, or average standard drinks consumed per occasion. For recurrent attacks of pancreatitis, young age (<35 years in Patra et al., <40 years in Lankisch et al.) was found to be the most significant risk factor statistically [28].

The plausible reasons explaining the association of Desi Daru/IMIL with acute necrotising pancreatitis are the presence of varying alcohol content (as high as 70%) and the presence of harmful impurities in addition to ethanol, such as long-chain alcohols (fusel alcohols), aldehydes, methanol, and lead. The concentration of these impurities can reach toxic levels due to poor quality control and inadequate distillation. Based on the "critical mass hypothesis," these impurities can enhance the oxidative stress of ethanol and can decrease the threshold for developing acute pancreatitis [29]. Due to the cheap price, daily drinkers of the elemental occupation group prefer Desi Daru/IMIL, which explains the study finding of a significant association between the type of alcoholic beverages and occupation groups and drinking patterns.

Limitations

This study is cross-sectional, examining the prevalence of pancreatitis at a certain point in time, and cannot evaluate incidence or analyse associations. Secondly, confounding factors were not studied, such as diabetes, obesity, and smoking. Thirdly, the data on alcohol intake is derived from patient self-reports and recollections. There may be an underreporting of alcoholic years, the amount of alcohol consumed on each occasion, and the number of neglected pain episodes. Fourth, the ethanol/alcohol concentration in Desi Daru/IMIL ranges significantly from 28% to 70%, with a standard value of 40% used for uniform calculations. Consequently, the threshold for pancreatitis may be surpassed more readily with the consumption of higher potency Desi Daru/IMIL, which is not considered. Furthermore, all patients consuming Desi Daru/IMIL are consolidated into a single group. Fifth, GMCH Nagpur is a tertiary care centre in Central India in which patients not managed/manageable at primary/community healthcare centres are also referred for further care. Therefore, the prevalence of acute necrotising pancreatitis and heavy drinkers can be high. And lastly, GMCH Nagpur is a government-based Central India institute that is used mainly by middle and lower socioeconomic class patients, so the prevalence of Desi Daru/IMIL drinkers or elementary occupationers can be high. Therefore, the generalizability of the findings from this study requires further investigation.

## Conclusions

In conclusion, drinking patterns are found to be statistically significantly related to occupation groups and average standard drinks consumed per occasion. The type of alcoholic beverages is statistically significantly related to occupation groups, drinking patterns, and average intake years. On initial presentation, the type of acute pancreatitis is statistically significantly related to the type of alcoholic beverages and drinking patterns. Therefore, daily drinking of Desi Daru/IMIL has a higher chance of acute necrotising pancreatitis, whereas daily drinking of distilled alcohol has a higher chance of acute interstitial pancreatitis on initial presentation.

Alcohol consumption is a social activity; the results of this study may be affected by many confounding factors. This study demonstrates that no safe drinking threshold exists for acute pancreatitis, making it imprudent for non-drinkers to start drinking.
